# Testing the role of spontaneous activity in visuospatial perception with patterned optogenetics

**DOI:** 10.1371/journal.pone.0318863

**Published:** 2025-02-27

**Authors:** Kengo Takahashi, Samuel Pontes Quero, Julien Fiorilli, Davide Benedetti, Rafael Yuste, Karl J. Friston, Giulio Tononi, Cyriel M.A. Pennartz, Umberto Olcese, TWCF: INTREPID Consortium

**Affiliations:** 1 Swammerdam Institute for Life Sciences, University of Amsterdam, Amsterdam, Netherlands; 2 Amsterdam Brain and Cognition, University of Amsterdam, Amsterdam, Netherlands; 3 Department of Biological Sciences, NeuroTechnology Center, Columbia University, New York City, New York, United States of America; 4 Wellcome Trust Centre for Neuroimaging, Institute of Neurology, University College London, London, United Kingdom; 5 Department of Psychiatry, University of Wisconsin-Madison, Wisconsin, United States of America; PLOS: Public Library of Science, UNITED KINGDOM OF GREAT BRITAIN AND NORTHERN IRELAND

## Abstract

A major debate in the field of consciousness pertains to whether neuronal activity or rather the causal structure of neural circuits underlie the generation of conscious experience. The former position is held by theoretical accounts of consciousness based on the predictive processing framework (such as neurorepresentationalism and active inference), while the latter is posited by the integrated information theory. This protocol describes an experiment, part of a larger adversarial collaboration, that was designed to address this question through a combination of behavioral tests in mice, functional imaging, patterned optogenetics and electrophysiology. The experiment will directly test if optogenetic inactivation of a portion of the visual cortex not responding to behaviorally relevant stimuli will affect the perception of the spatial distribution of these stimuli, even when the neurons being inactivated display no or very low spiking activity, so low that it does not induce a significant effect on other cortical areas. The results of the experiment will be compared against theoretical predictions, and will provide a major contribution towards understanding what the neuronal substrate of consciousness is.

## Introduction

In the past couple of decades several theories have emerged to explain how the brain can generate consciousness [1], also often defined as what it feels like to experience sensory stimuli, memories, etc. [2]. Currently, most data either supporting or challenging theories of consciousness comes from a combination of sensory stimulation paradigms, behavioral tasks and neural recordings [3]. All this has greatly advanced our understanding of which brain areas and patterns of activity correlate with consciousness – and in particular with perceptual awareness [4,5]. However, the inability to perturb neural activity in a precise fashion in human experiments precludes the possibility to go from the level of correlation between brain activity and consciousness to that of causation [6]. Techniques such as optogenetics now allow unprecedented possibilities to manipulate neuronal activity in animal models [7–9]. Nevertheless, even in the case of animal experiments designed to test the neural origins of key aspects of consciousness such as conscious access, mostly correlative data has been collected so far [10]. One reason underlying this fact pertains to the lack of precise, circuit-level mechanistic explanations that most theories of consciousness provide about how the brain would be able to generate consciousness. In fact, while optogenetics and similar approaches allow to precisely manipulate neuronal circuits, theories often fail to discuss exactly how such manipulations should take place [1].

To address this, we designed a set of experiments capable of testing the mechanistic link hypothesized to exist between neuronal activity and consciousness according to three theories of consciousness: Integrated Information Theory (IIT), Neurorepresentationalism (NREP) and Active Inference (AI-C). Such a link will be here briefly discussed for each theory, after which we will describe the experiment that we will perform to address these theoretical predictions.

The Integrated Information Theory (IIT) posits that a fundamental relationship exists between the quality and quantity of consciousness, and the causal structure engendered by a system’s constituent mechanisms (neural or otherwise) [11,12]. This causal power, intrinsic to the system, arises from the interplay between a system’s components. IIT predicts that modifications to the causal structure at a relevant spatiotemporal scale, independent of constituent changes in activity state, should manifest as changes in perceptual correlates, or alterations in conscious experience. Recent elaborations of the IIT framework have yielded predictions concerning the physical substrate of spatial experiences [13]. Specifically, IIT predicts that the perception of spatial extension corresponds to particular types of causal structures generated by grid-like networks, such as those observed in retinotopic cortices. IIT postulates that even inactivating (or physically removing) a substantial number of currently inactive neurons within these grid-like structures would modify the causal edifice they specify, thereby altering the experience of spatial extendedness. Thus, in the context of this proposal, IIT offers a counterintuitive prediction: the (externally induced) inactivation of a large population of presently inactive (i.e., silent) neurons in retinotopic cortices would lead to a corresponding reduction in perceived space.

Set within the overarching framework of predictive processing (PP) [14–17], NREP posits that perception emerges from the construction of inferential representations at both high and low levels of brain organization. These representations can be construed as perceptual hypotheses that are continuously refined through interaction with bottom-up sensory inputs. This interaction serves to update generative models representing the underlying causes of these sensory changes [18,19]. The link to consciousness arises when high-level, multi-area representations are generated, encompassing a spatially extensive and multimodally rich portrayal of the subject’s current environment, whilst these high-level representations simultaneously correspond to low-level neural representations concerned with small-scale, unisensory details of percepts [18,19]. To account for the qualitative richness of these high-level representations, NREP emphasizes the significance of not only bottom-up and top-down cortical connectivity but also lateral (intermodal) connectivity. This perspective leads to the postulate that, next to stimulus-induced changes in firing rate, background activity in neuronal networks is necessary for the generation of consciousness, even within brain regions and neuronal groups not directly involved in processing specific object features [18–20]. For instance, background activity in non-visual sensory cortical systems is important for visual perception. This background activity extends to brain areas mediating modality- and sensory-specific identification of the perceived feature, encompassing not only other sensory cortices but also higher associative areas (e.g., parietal cortex) and potentially even motor cortex [21].

Finally, AI-C, a theory also grounded in predictive processing, emphasizes the role of inference of action policies aimed at minimizing expected, rather than current, prediction error [22–27]. In simpler terms, active inference involves covert or overt sampling of sensory information to reduce uncertainty regarding the source of sensations. Overt sampling in the visual domain primarily manifests through eye movements or other physical movements. Covert active inference, on the other hand, refers to the act of attending to (or ignoring) specific features of an object or its location. This framework encompasses attentional capture, such as the involuntary shift of spatial attention towards a salient stimulus. Notably, AI-C refrains from making specific hypotheses regarding neuronal activity beyond those encompassed by the broader predictive processing framework [15]. However, AI-C diverges from NREP by focusing on action and action policies as being necessary for consciousness (whereas NREP does not), whereby expected prediction errors are minimized through action policy inference. In contrast, NREP emphasizes minimizing current prediction errors via inference on present sensory inputs.

The experiment described here will test the different predictions that IIT, NREP and AI-C make with respect to the role in the generation of consciousness played by background neural activity (NREP, AI-C) or lack thereof (IIT), meant as the spontaneous level of activity displayed by neurons not directly activated by the sensory features that constitute the object of perception. Consciousness will be operationalized by studying the experience of visuospatial extendedness. Briefly, IIT postulates that not only active, but also inactive (i.e., silent) neurons contribute to conscious experience [11,13]; specifically, neurons in retinotopic areas would contribute to visual perception not only when displaying visually evoked responses, but also when not firing action potentials at all. Therefore, inactivating both active as well as inactive neurons (i.e., making them non-activatable; e.g., by cooling, reversible lesions, or other methods such as optogenetics and optochemistry) should disrupt the cause-effect structure of a neural system, and consequently modify the quality of consciousness. In particular, the inactivation of a large number of inactive neurons in retinotopic cortex (ideally all inactive neurons located in primary visual cortex V1 and higher order visual areas; HVAs) [28] during the ~200 ms time window following stimulus onset that is required for the neural processes underlying the perception of a visual object to emerge [29–31] (but before any report of such perception is made [5,32]), is expected to induce a collapse in experienced visual space – similar to hemineglect or hemianopia. In contrast to IIT, NREP and AI-C postulate that only active neurons contribute to conscious experience. Inactive neurons do not contribute to perception because they exert no effect (i.e., cause no activity changes) on the rest of the system, and thus inactivating an already inactive neuron should not affect perception. The belief updating in PP is mediated by active neurons. However, a differential prediction is made in this respect by the two forms of PP under scrutiny here. NREP assumes that, in addition to stimulus-responsive neurons, background activity in the network of brain areas mediating visual perception [21] is necessary for conscious experience, as long as spontaneous levels of activity are strong enough to exert causal influences on connected neurons [18,19]. Thus, only inactivating those neurons that are truly inactive (i.e., do not send spikes to other neurons), or whose spiking activity is so weak that it does not significantly modulate the activity of other neurons (i.e., virtually inactive), will not have an effect on perception [18,19]; inactivating a sufficient amount of neurons firing at baseline level should instead disrupt perception if this baseline activity affects other areas. In AI-C, instead, neurons with a low background level of activity may or may not contribute to conscious experience; they might be detrimental to it, by decreasing confidence in a precise sensory input [22] or paradoxically improve discrimination (by evincing a form of representational sharpening). Thus, AI-C predicts that inactivating background activity in the visual system would lead to a change in perception (in terms of psychometrics), but is non-specific about how perception would change [22,23,33].

### Current study

The differential predictions of the three theories are here worked out in the setting of spatial experience, quantified by studying distance estimations at the psychophysical level in mice, that we will then attempt to modify via specific optogenetic distortions of the neuronally constructed map of visual space.

**Fig 1:**
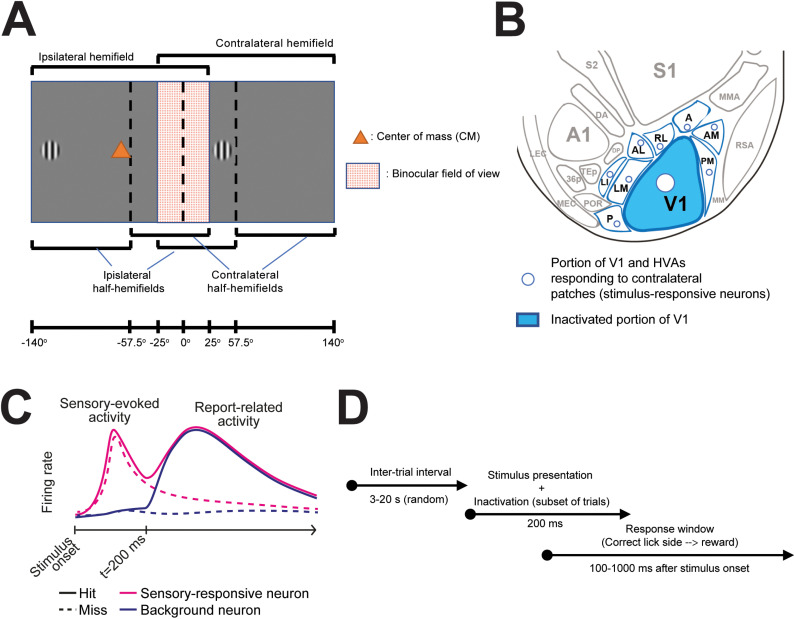
Experimental outline. **A.** Two Gabor patches will be simultaneously displayed in the left and right monocular fields of view. Mice will have to determine whether the center of mass between the two Gabor patches falls in either the left or right hemifield of view and lick towards the corresponding reward spout to receive a reward. In this figure it is assumed that both recordings and optogenetic manipulations will be performed in the left cortical hemisphere. Hence, the left visual field will be the ipsilateral one, while the right visual field will be contralateral. Gabor patches will never be displayed in the binocular field of view (red-textured area). Note that the stimulus presentation time is shorter than the onset of saccades in mice, therefore we do not expect the borders between monocular and binocular cortex to change significantly during stimulus presentation. **B.** Patterned illumination will be used to achieve the optogenetic inactivation of the portion of V1 that does not respond to the visual stimulus (blue areas outside the circles), as demonstrated through imaging experiments. White circles correspond to the cortical locations showing stimulus-evoked responses in both V1 and HVAs. The region of V1 being illuminated will comprise a ring of ~250 μm of tolerance, in order to account for dendritic arborizations of neurons in the non-inactivated portions of V1, and prevent the risk of reducing any input such regions might receive from surrounding regions in VI and NB conditions [60]. We will not inactivate HVAs in view of the high spatial resolution that would be required to selectively target non-responsive patches of each individual HVA.Areas P, LI, LM and AL are HVAs that are considered to be part of the mouse ventral stream. Areas RL, A, AM and PM are HVAs that are considered to be part of the mouse dorsal stream [61]. For all areas indicated in the panel, standard acronyms are used [54,61]. **C.** Schematic representation of the expected time course of neuronal activity during hit (solid lines) and miss (dashed lines) trials for neurons responding to the displayed Gabor patches (pink) or not (blue). Note the widespread emergence of report-related activity starting from about 200 ms after stimulus onset [31,50]. **D.** Timeline of the behavioral task. After a variable inter-trial interval, sensory stimuli will be displayed for 200 ms. In a fraction of trials, inactive portions of V1 will be optogenetically inactivated. Mice will be rewarded in case of a correct response, if licking is recorded during the response window, lasting from 100 ms to 1000 ms after stimulus onset.

Briefly, head-fixed mice will be trained to report whether the spatial center of mass of a pair of visual patches falls in the left or right hemifield of view (Fig 1A) by licking towards a reward spout either on the left or right side; correct actions will be rewarded by a drop of milk or juice. This task requires mice to estimate the relative (and not just the absolute) position of the presented visual stimuli, and has been designed to specifically test the theories’ different predictions about the neuronal mechanisms enabling visuospatial perception. Patterned optogenetics via inhibitory opsins will then be used to hyperpolarize (and thus inactivate) neurons in portions of V1 (left hemisphere only) not displaying sensory-evoked responses during the first 200 ms after stimulus onset (before any widespread motion and report-related activity is observed in V1 [31]) – Fig 1B-D. The main experimental readout will be a change in behavioral response (reflecting a functional hemineglect) upon the inactivation of already inactive (or sensory unresponsive) V1 neurons in one cortical hemisphere showing a background level of activity (i.e., no direct sensory-evoked responses). IIT predicts that, even if portions of the visual cortex responsible for processing visual stimuli were active and fully functional, inactivation of the inactive parts of V1 (i.e., all portions of the visual cortex not involved in stimulus processing) would prevent spatial experience. This means that mice would not be able to assign a spatial location, and consequently assess the relative position, of visual patches presented in the hemifield of view contralateral to the optogenetically inactivated hemisphere. This would correspond to a functional hemineglect: visual information presented in the “neglected” hemifield will no longer be usable to solve the center-of-mass task, although a residual form of perception about the presence of visual stimuli may remain (but this cannot be tested in the current protocol). For NREP, such functional hemineglect would only occur when inactivating neurons with a sufficient level of background activity, while the optogenetic inactivation of inactive or virtually inactive neurons should not induce a behavioral change. Finally, AI-C predicts no functional hemineglect, but rather changes in behavioral responses, such as faster reaction times and/ or either enhanced or worsened psychometrics as measured by threshold, slope, etc.

The proposed experiment is based on the premise that spontaneous, not sensory-evoked activity in visual cortex can be modulated or titrated to different levels, that are required to test the different predictions of the three theories under scrutiny. Specifically, we have defined the following three activity levels:

-Minimally active (MA): This is to be understood as the lowest level of activity that can be observed in visual cortex. This will be quantified as the average number of action potentials observed in the ~200 ms time window after stimulus onset during which perception is expected to take place according to the three theories (and before the emergence of widespread report-related activity in V1 and HVAs [31]) in neurons not directly activated by visual stimuli, and as the proportion of neurons – out of all neurons not directly activated by visual stimuli – not firing any action potential during any given trial in the same temporal window. More precisely, the MA level will be defined as a condition during which spontaneous neuronal activity is statistically indistinguishable from zero firing rate.

-Virtually inactive (VI): the highest level of spontaneous firing activity that does not result in a significant change in the activity of downstream regions (i.e., cortical areas monosynaptically targeted by V1. The VI level would ultimately correspond to a condition in which neurons not activated by sensory stimuli are not silent, but still their activity is so low that it does not drive, at the population level, other monosynaptically connected regions. This level will be determined via measuring the variation in the activity of areas downstream to V1 following optogenetic inactivation of V1. Values of spontaneous V1 activity for which no statistically significant change in downstream activity can be observed after optogenetic V1 inactivation will therefore be classified as VI.

-Normal background (NB): This corresponds to a level of spontaneous activity that is present in neurons not directly activated by visual stimuli (in the already mentioned ~200 ms window after stimulus onset), and that is higher than the threshold for the VI condition. Values of spontaneous V1 activity for which a statistically significant change in downstream activity can be observed will be classified as NB.

**Fig 2:**
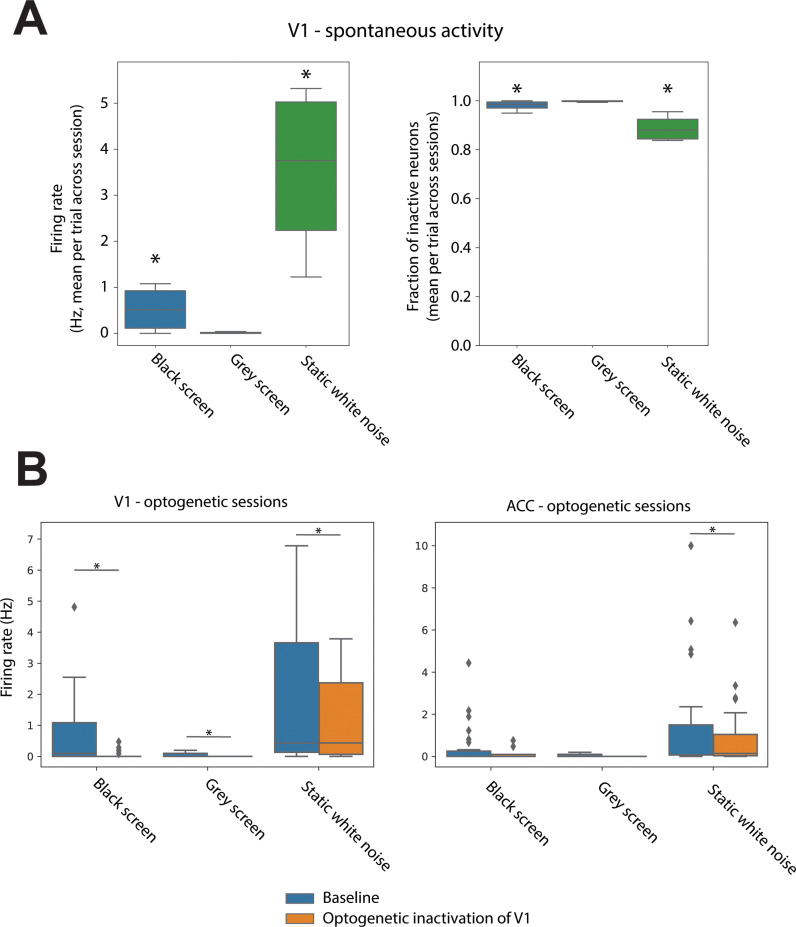
Pilot results. Dual-area Neuropixels 1.0 probe recordings [44] were collected during 5 recording sessions from two head-fixed mice, habituated to head-fixation and to the presentation of different visual stimuli over a period of several weeks. Additionally, during inter-trial periods mice were exposed to a gray background, to which they became habituated. Total number of identified single neurons following spike sorting with Kilosort 2.0 [56]: 137 neurons in V1 and 95 neurons in the anterior cingulate cortex (ACC). Mice expressed the inhibitory opsin iC++ [62] in pyramidal neurons in ACC. This was achieved via viral injection of an AAV vector mediating the expression of iC++ under the CaMKII promoter. **A.** The activity of V1 units was quantified as a function of which visual background was presented (black screen, grey screen or static white noise pattern – grey-valued bins – shown for periods of several minutes). Recordings were subdivided into non-overlapping windows of 200 ms, during which we computed the average firing rates of detected neurons (left), and the fraction of neurons not displaying any action potential during each 200 ms window (right). We found that, only when displaying a grey visual background activity in V1 was not significantly higher than 0 for both metrics. Asterisks indicate values significantly different from either 0 (firing rates) or 1 (fraction of inactive neurons) (p < 0.05, one-sided t-test). **B.** The effect of optogenetic inactivation of V1 (over 200 ms windows) was compared for both V1 and ACC as a function of visual background. Optogenetic inactivation of V1 was effective in reducing V1 activity across all visual backgrounds. However, a signification reduction was observed in ACC, a downstream area to V1, only when displaying a static white noise. Asterisks indicate significant differences between baseline firing rates and epochs with optogenetic illumination (p < 0.05, paired t-test); note that a smaller dataset was used in panel B compared to panel A. Taken together, these results indicate that the use of a grey background induces a level of spontaneous activity in V1 compatible with the MA level, i.e., statistically indistinguishable from 0; the use of a black background instead elicits some spontaneous activity, but at a level that is not high enough to make a difference to downstream areas – and thus in line with the definition of the VI level. Finally, the use of a random pattern as a visual background further increases spontaneous V1 activity to a level compatible with the NB level.

These three different conditions will be achieved by modifying the visual background shown to mice (e.g., black, gray or white noise background), and by monitoring spontaneous fluctuations in baseline activity, for instance as a consequence of arousal, as measured in terms of pupil size [34,35]. Based on these aspects, we aim to subdivide experimental epochs into one of the three activity levels. The subdivision was carried out a priori relative to the displayed visual background, and a posteriori with respect to the arousal level. Fig 2 shows the results of pilot experiments meant to verify the feasibility of achieving all three different levels.

By performing optogenetic inactivation of portions of V1 not displaying sensory-evoked responses in mice performing the center-of-mass task, and by comparing the results obtained across the three levels of spontaneous activity, we expect to be able to provide sufficient evidence in support of one out of the three theories under scrutiny. Details about the specific prediction of the three theories for each activity level can be found in the section “Operational Hypotheses”.

### Timeline, status and pilot experiments

The experiment described here is part of a larger adversarial collaboration in which IIT, NREP and AI-C will be compared via a series of experiments. Following a successful set of pilot experiments that demonstrated the possibility of obtaining all three planned levels of spontaneous activity (Fig 2), a consortium led by one of the authors (CMAP, University of Amsterdam) was awarded a grant funded by the Templeton World Charity Foundation (TWCF number: 0646; https://doi.org/10.54224/20646). The peer-reviewed grant application was pre-registered on OSF (https://osf.io/35rhx) and later updated to reflect the results obtained in a further set of pilot and preliminary experiments (https://osf.io/4rn85). For each of the experiments composing the larger adversarial collaboration, a protocol paper is to be published, based on the latest version of the pre-registration and on the most recent results from preparatory experiments. The experiments described here thus faithfully reflect the pre-registered project. Experiments are to be performed in parallel at two sites: the lab of Umberto Olcese at the University of Amsterdam (herein referred to as experimental lead) and the lab of Rafael Yuste at Columbia University (herein referred to as replication lead). All details about the replication efforts are described in the following sections. Currently, following an extensive set of preliminary experiments, data collection is at an early stage.

## Materials and methods

### Participants

Experiments will be done in mice (C57BL/6J background) expressing the GCaMP6 calcium indicator in a subset of cortical neurons. In the experimental lab, we will use the Rasgrf2-dCre x Ai148D line, in which GCaMP6f is expressed mainly in layer 2–3 cortical neurons following exposure to the antibiotic doxycycline (Jax #030328; Jax #022864) [36]. These experiments have already been approved by the Dutch Commission for Animal Experiments and by the Animal Welfare Body of the University of Amsterdam, under license AVD11100202215932. Experiments conducted in the replication lab will use the mouse line Slc17a7-IRES2-Cre x Ai162 (Jax #023527; Jax # 031562), which constitutively expresses GCaMP6s in excitatory neurons. All the animal procedures at the replication lab were approved by the Columbia University Institutional Animal Care and Use Committee (IACUC), in compliance with the relevant National Institutes of Health guidelines, under protocol AC-AABN3562. Mice will be 6–8 weeks of age at the beginning of handling and training procedures. Training will take place over a period of max 12 months. Both male and female mice may be used. Mice will be group housed and kept on a reverse day-night schedule; in case this is not possible, all experimental procedures will be done in the dark period or as close as possible to it. Mice will have ad libitum access to food, even though a water restriction regime will be implemented to motivate animals for the behavioral task. Mice will receive at least 25 ml/kg/day of liquid, and will be monitored so that, on every given week, their weight does not decrease below 90% of the weight measured during the previous week. All experiments have been designed in a way that minimizes animal suffering. First of all, data collection will stop as soon as a sufficient amount of data will be collected (see the next section). Second, we will take all possible measures to alleviate animal suffering, as long as these are compatible with experimental objectives. First of all, analgesia and anesthesia will be employed when necessary (see the section on “Surgical procedures”). Second, animals will be group housed and cage enrichment will be provided. Finally, we will make sure that any consequence of water restriction will be limited (see earlier in this section for details).

### Sample size and exclusion criteria

Based on the level of background activity in the patches of visual cortices (primary visual cortex V1) not directly activated by visual stimuli, we will subdivide experimental sessions in 3 distinct types: minimally active (MA), virtually inactive (VI) and normal background activity (NB). In order to compute sample size, we have estimated that mice will achieve a performance corresponding to 70% correct responses, and that these should drop below 30% to establish that optogenetic inactivation was effective in significantly changing behavioral responses. This is a level in line with the highest false alarm rate typically observed, which would correspond to the expected response rate in case of hemineglect. For hit rates, we estimate a standard deviation of 20% across sessions. This corresponds to a large effect size (which we conservatively set to 0.8). Whether a sufficient sample size is reached will be estimated in the course of the experiments via a Bayesian sequential stopping rule [37,38]. Setting the Bayes factor BF_10_ to larger than 3 (moderate evidence), we estimate that we will have to collect data from 11 experimental sessions (median value) for each of the three session types with different levels of background activity (MA, VI and NB) in order to have sufficient data to assess the hypothesis that inactivating inactive neurons impairs spatial experience (H1). Analogously, the stopping criterion for the null hypothesis (H0: no effect of inactivating inactive neurons) has been set to BF_10_ < ⅓; this corresponds to a median sample size of 12 sessions. Experiments will stop when, a value BF_10_ > 3 or BF_10_ < ⅓ is obtained, separately for each of the three levels of background activity (MA, VI and NB). Alternatively, experiments will terminate after the collection of 21 experimental sessions, for each of the three levels of background activity, which corresponds to a chance greater than 75% of obtaining BF_10_ > 3 or BF_10_ < ⅓. Moreover, we include an additional condition holding that the experimental sessions need to be acquired in at least 4 distinct mice, to prevent excessive statistical dependencies between sessions. Importantly, this is a minimum number of mice that we plan to use in recording sessions, and we aim to perform experiments in more than 4 mice (at least 6), both in the experimental as well as in the replication lab. Nevertheless, more mice are expected to successfully undergo all experimental procedures (training, surgeries, etc.) before reaching the final experimental stage.

No analysis meant to assess experimental outcomes will be conducted on the data prior to completing all data collection, except for those analyses aimed at: a) ensuring animals do not meet the exclusion criteria (see below), b) verifying that collected data (electrophysiology, imaging) is of sufficient quality, c) verifying that opsin expression and optogenetic inactivation have been successful, and d) enabling experiments to be correctly performed. We will stop collecting the data upon reaching the stopping criterion or, alternatively, the predefined maximum sample size, when taking into account experiments not meeting any of the exclusion criteria.

#### Exclusion criteria.

A mouse will be excluded if:

- It fails to learn the center of mass task (see the later section “Behavioral procedure”) sufficiently well (hit rate <  60% – i.e., proportion of licks to the correct side, left or right – for stimuli with center of mass falling in all tested locations) or it shows an excessive false alarm rate ( > 20–30%, defined as the proportion of probe trials without visual stimuli during which mice lick towards one of the two reward spouts);
- Post-mortem histological analyses show that opsin expression was not covering the full monocular portion of V1.

An experimental session will be excluded if:

- Widefield calcium imaging is of insufficient quality to allow optogenetic targeting;
- Task performance during trials without optogenetic inactivation is of insufficient quality (hit rate <  60% for stimuli with center of mass falling in the tested locations, or false alarm rate >  30%);
- Electrophysiological recordings (if performed) and arousal monitoring indicate that optogenetic inactivation was not able to reduce spiking activity in the illuminated regions below the threshold identified as maximum activity for MA neurons, or that a specific level of background activity (MA, VI or NB) could not be achieved.

### Surgical procedures

**Fig 3:**
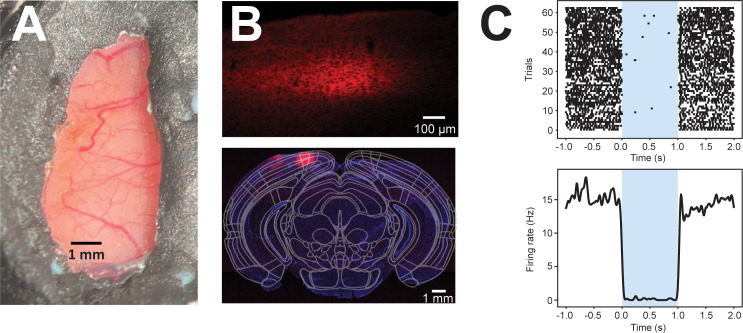
Cranial window and optogenetic inactivation. **A.** Example of PDMS window placed on the left hemisphere. Dental cement (grey) is used to stably fix the window onto the remaining skull. **B**. Example histological sections (top: 10X magnification; bottom: 5X magnification, coronal section located 4.20 mm posterior to bregma) showing the expression of the red-shifted inhibitory opsin Jaws in the visual cortex of a pilot animal. In the final experiment we will perform multiple viral injections in GCaMP-expressing mice to cover the entirety of V1. **C**. Example neuron showing Jaws-mediated inactivation upon illumination with a 637 nm laser (shaded area). Top: raster plot. Bottom: Average peri-event time histogram (PETH) ± SEM.

Mice will be implanted with a custom-made head bar. In the same surgical procedure, we will replace the dorsal part of the left skull and replace it with a custom-made Polydimethylsiloxane (PDMS) transparent window (Fig 3A) [39], which will enable to perform both neuronal imaging as well as optogenetics. About 3 weeks before the planned start of experimental sessions (i.e., when training will be completed), we will inject a combination of viral strains mediating the pan-neuronal expression of the red-shifted inhibitory (chloride-dependent) opsin Jaws (ssAAV-8/2-hSyn1-Jaws_KGC_tdTomato_ER2-WPRE-bGHp(A) [40] – Fig 3B-C; see also later sections for alternative strategies). Injections will target V1. Based on previous experiments, an injection volume of about 0.5 μl should be sufficient to cover all required cortical surfaces. At the end of the experiment, small openings will be made in the PDMS window to enable electrophysiological recordings [41]. All surgical procedures will be done under isoflurane anesthesia (induction at 3%, maintenance at 1.5%–2%). The analgesic agent Carprofen (5 mg/kg) will be provided on the day of the surgery and for two subsequent days.

### Visual stimuli

Visual stimuli will consist of Gabor patches (about 10 ± 2 deg diameter, displaying static gratings at 0.04 cycles per degree, 100% contrast). Two patches will be shown, one in the contralateral and one in the ipsilateral field of view (Fig 1A). Stimuli will only be shown in the portions of the monocular visual fields covered by the screen ( > 25 deg and <  109 deg from the midline (Fig 1A and Fig 5B). The precise locations of the visual stimuli will be discussed in the next sections. For the purposes of considering whether the center of mass of the presented stimuli falls in the contralateral or ipsilateral half of the hemifield – something that is important when considering the predictions of the various theories – the midline of the partial field of view encompassing the binocular and one monocular field (i.e., the portion of the visual field being accessible by one cortical hemisphere via direct thalamocortical projections) will be considered (approximately 57.5 deg from the midline, Fig 1A – see also Fig 5B for further details about the actual locations of Gabor patches. The elevation of the Gabor patches will not be behaviorally relevant and will be kept at the level of the animals’ eyes. In a subset of control trials, we will only display one Gabor patch, to measure baseline localization accuracy.

#### Background stimuli .

The visual background on which the Gabor patches are shown will be varied in a way that allows to titrate the baseline activity of neurons in V1 according to one of the three levels: MA, VI and NB. Based on the results obtained in pilot experiments, we will use a grey background for the MA condition, a black background for the VI condition, and a static white noise background for the NB condition (Fig 2). Based on electrophysiological recordings performed during actual experiments, we may however have to change the use of visual backgrounds to reach a particular level of background activity, if for example we observe that the association between visual background and the level of spontaneous baseline activity varies between animals or recording sessions.

### Behavioral procedure

Animals will be trained to discriminate whether the center of mass of the displayed Gabor patches lies in the left or right hemifield of view (left or right of the midline). Details about the training procedure have been devised in the course of pilot experiments. Animals will respond by performing licks towards either a left or right lick spout. Correct responses will be rewarded by a drop of milk. Licks will be monitored starting from 100 ms after stimulus onset, to account for physiological reaction times (lick latencies). Stimuli will be shown for 200 ms, based on the shortest temporal presentation window that pilot mice needed to perform the center of mass task. Based on recent studies, this window is expected to last from stimulus onset until at most the estimated onset of report-related activity in V1 [31]. Rewards will be delivered immediately after a correct lick is detected. Inter-trial intervals will be randomly taken from an exponential distribution with a mean of six seconds (minimum 3 and maximum 10 to 20 seconds; the exact values will be chosen on a per-mouse basis to maximize the number of trials performed in every experimental session). Inter-trial intervals will not differ based on trial outcome (correct, incorrect response or miss). In a subset of probe trials, we will not show any stimulus (to enable the assessment of the false alarm rate).

#### Training procedure and location of visual stimuli .

Mice will first be habituated to experimenters and training setups. A drop of milk will be initially awarded from lick spouts at any moment when mice lick in front of the grey screen. Once mice learn the association between lick spout and reward, we will unilaterally present a Gabor patch (size: ~30 deg, spatial frequency: 0.04 Hz) on the right side of screen (location: 90 deg vergence angle) for ~4 seconds in each trial. Mice will have to lick during stimulus presentation to receive a reward. We will continue this step until the hit rate becomes higher than 70% in one session. We will compute the hit rate based on the first lick after the stimulus onset after the100 ms grace period. Then, we will start showing a smaller Gabor patch (size: ~25 deg) unilaterally either on the left or right side of the screens until the hit rate exceeds 70% in one session. Once mice can reliably maintain this hit rate (approximately 3–4 sessions), we will start displaying a smaller Gabor patch (size: ~15 deg) randomly in one of six different unilateral locations (approximately −106, −70, −34, 34, 70, and 106 deg vergence angles). These locations were chosen as representative of three locations spanning the full monocular field of view (to make sure mice pay attention to the full field of view and do not develop a behavioral strategy based on focusing on a single spot on the screen): the outermost part and innermost part of the screen, as well as the in-between middle point (Fig 5B). In order to prevent the innermost Gabor patches from falling into the binocular field of view, they will be placed at the middle point between the edge of the monocular zone and contra/ipsilateral half-hemifield. A depiction of the chosen locations is presented in Fig 5B. We aim to obtain >70% hit rate overall but minimum >60% hit rate in each condition to ensure comparable performance among different locations.

Following the unilateral configurations, the center of mass task will be introduced, starting from easy bilateral configurations. One Gabor patch (expected size: ~10 ± 2 deg) will be presented in the monocular visual field (location: approximately −106 or 106 deg vergence angle) and the other (expected size: ~10 ± 2 deg) in the binocular visual field (Location: approximately −8 or 8 deg vergence angle). Each session will consist in principle of approximately 64% of trials with the center of mass configurations (32% on each side) and 36% of trials with the unilateral configurations (6% each on the six different locations); different proportions of trials may be chosen based on the performance of individual mice. We aim to obtain >70% hit rate overall, but minimum >60% hit rate in each condition. Once mice learn these easy binocular configurations, more difficult binocular configurations will be introduced, such as the combination between −106 and 25 deg or −25 and 106 deg. In the end, the final configurations (left center of mass: approximately −106 and 34 deg; right center of mass: approximately −34 and 106 deg) will be introduced. Upon >70% hit rate for the binocular configurations as well as > 60% hit rate in each condition, the stimulus presentation time will be reduced from 2.5 s to 200 ms. Pilot experiments have been performed to verify that mice can learn to perform the center of mass task. Mice were able to report with > 60% accuracy the location (left vs. right) of Gabor patches, both when a single patch was presented in one of the planned locations along the horizontal axis, as well as in a simple center of mass configuration (one peripheral and one central location).

### Data collection procedures

#### Retinotopic mapping and widefield imaging .

In order to locate the visual cortical areas that are active/inactive during the task, we will initially perform retinotopic mapping to determine the boundaries of V1, followed by test presentations of the task stimulus to pinpoint areas within the global visual cortical map that are activated by the Gabor patches that will be used in the final experiment (Fig 5). Briefly, we will show a flickering black and white checkerboard stimulus drifting in different directions (for parameter details, see [42]) on a screen (width =  36 cm, height =  21 cm), placed at the distance of 11 cm from the eye to the closest point of the screen in the right visual field. Concurrently, cortical responses to violet (405 nm, used to monitor hemodynamic responses) and blue (470 nm, used to elicit GCaMP6 fluorescence) LED stimulation will be recorded using a custom made widefield microscope at sampling frequency of 30 Hz with twofold pixel binning [43]. The mapping procedure will be performed after training and prior to the experimental period. The borders of V1 will be identified by computing the sign map derived from the altitude and azimuth maps indicating retinotopic responsiveness in the visual cortex [42]. V1 will be identified based on the reversal in the sign map that occurs at the border between V1 and HVAs [42].

Presentation of the task relevant stimuli will be performed with the exact same parameters as the experimental sessions (size, locations, background activity) and recorded similarly. The obtained maps and active areas will be registered to the vascular landmarks of the cortical surface to correct for differences in mouse position between experimental sessions.

#### Multi-area ensemble recordings.

Multi-area ensemble recordings will be done by performing small openings in the PDMS window over the area of interest, via which multi-channel silicon probes (Neuropixels 1.0 or 2.0 probes [44,45]) will be inserted. Detailed methods can be found in [31,41,46,47]. Briefly, the PDMS window covering the cortex will be punctured over the cortical areas of interest, after which one of more laminar probes will be inserted. After the experimental session has been completed, the probe will be retracted and the craniotomy closed with a silicon-based sealant. Multiple recordings will be acquired in the same mouse across subsequent days. A maximum of three recording probes will be simultaneously used. Data will be recorded with a 30 kHz sampling frequency.

#### T wo-photon calcium imaging .

Within the control experiments performed at the replication lab, two-photon calcium imaging will be used instead of multi-area ensemble recordings to monitor neuronal activity. Importantly, two-photon calcium imaging allows to identify both active and inactive neurons, and is thus complementary to electrophysiology (that only enables researchers to detect spiking neurons). Two photon imaging and either holographic 2-photon stimulation or LED single-photon stimulation will be performed. The optical system consists on a customized two photon laser scanning imaging microscope equipped with a spatial light modulator (SLM) to perform simultaneous population photostimulation as described before [9,48,49]. For imaging the GCaMP6f sensors, a Ti:sapphire femtosecond pulsed laser tuned at 940nm will be used. The scanned laser at the sample will produce a 300 x 300 µm FOV using a 25x objective. For holographic photostimulation, a low repetition (200 kHz ~  1 MHz) pulse-amplified laser, working at 1040 nm will be used. The beam will be expanded by two telescopes (1:1.75, f =  100 mm and f =  175 mm; 1:4, f =  50 mm and f =  200 mm) to fill the active area of the SLM (HSP512-1064, Meadowlark Optics). The typical imaging power will be less than 50 mW, and typical average power used for each illuminated spot will be 2–5 mW. Simultaneous imaging and photostimulation will be controlled by Prairie View software and a custom-made MATLAB application. For single photon stimulation experiments an optic fiber equipped with a cannula will be roughly positioned over the cortex at shallow an angle to avoid touching the objective. The fiber will be coupled to a 617 nm LED and typically a 2–5 mW power will be used.

#### Behavioural monitoring.

Licking actions will be monitored via piezoelectric detectors, connected to a custom-made electronic board (Arduino-based). An infrared camera will be used to monitor pupil size and orofacial movements throughout experimental sessions [31,50]. All timestamps will be synchronized.

### Optogenetic inactivation

Patterned illumination and optogenetic inactivation of the portion of V1 – in the left hemisphere only – not directly activated by visual stimuli will be achieved through the integration of a digital micromirror (DMD), scanned laser projector (Sony MP-CL1A laser projector) into the widefield imaging system used above for retinotopic mapping.

**Fig 4:**
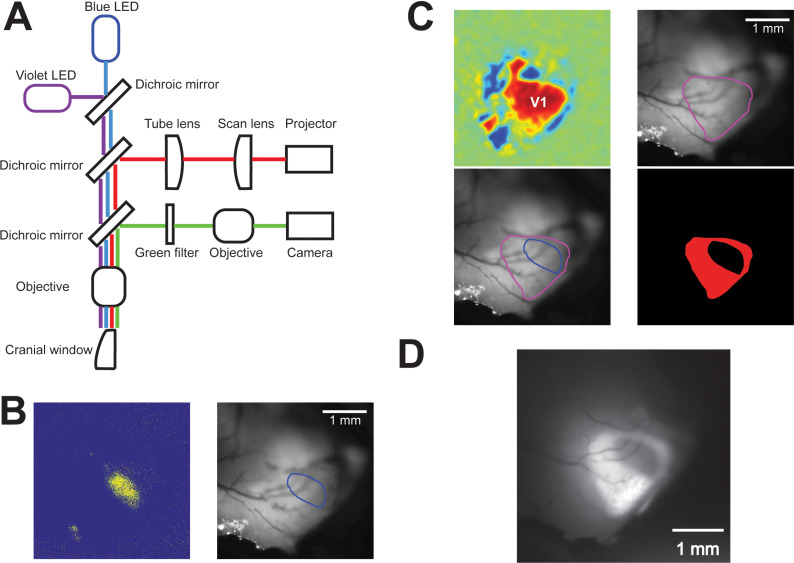
Patterned inactivation protocol. **A.** Optical path of the setup for simultaneous widefield calcium imaging (including monitoring of hemodynamic responses) and patterned illumination via a laser beam projector. Violet: LED light used to monitor the hemodynamic response (red path). Blue: LED light used to induce GCaMP6f fluorescence (green path). **B.** Widefield calcium imaging will be used to identify the portion of the visual cortex responding to Gabor patches presented in the experimentally relevant locations (Fig 5B). Left: widefield calcium response to a single Gabor patch, after correction for hemodynamic responses [43]. Right: outline of the visually responsive portion of V1 is computed and overlaid on the vasculature map for localizing it. **C.** Top-left: A sign map [42] is computed to identify the extension of V1 (data and code obtained from https://github.com/zhuangjun1981/NeuroAnalysisTools). Top-right: The outline of V1, as identified by the sign map, is overlaid on the vasculature map. Bottom-left: Overlay of the outline of V1 and of the portion of V1 responsive to the presented Gabor patch (see panel B). Bottom-right: The mask for patterned illumination is computed as the negative image of the portion of V1 responding to a Gabor patch. Red light is used to activate the inhibitory opsin Jaws. **D.** The final illumination pattern (see panel C) is projected on the cortex for inactivation the portion of V1 not responding to the presented Gabor patch.

Following retinotopic mapping, we will compute a set of configurations for the DMD for each of the visual stimuli that we will show in a given experimental session. We will project light only on selected portions of V1 – see Fig 1. The portions of V1 to be inactivated will determine a mask (black in the areas not to be perturbed and red elsewhere in line with Jaws’ excitation spectrum [40] (Fig 4B-C), that will be sent to the DMD projector and displayed on the cortex. The scanned image will be conjugated to the backfocal plane of the objective lens through a 4f double lens relay system (Fig 4A). To determine the mapping between the projected image and the imaged widefield field of view (FOV), an affine transformation matrix will first be calculated based on linear regression with a set of defined locations projected to the FOV. This transformation will be applied to any image provided to the projector. Optogenetic inactivation will be performed during the presentation of visual stimuli, i.e., from stimulus onset to ~200 ms afterwards. The efficacy of optogenetic inactivation will be tested in control experiments, in which laminar probe recordings and optogenetics will be simultaneously done. This will allow to verify the experimental parameters (e.g., laser power) necessary to effectively inactivate neurons across both the surface and depth of V1. An average power calibration will be taken by measuring radiant flux at the sample of a homogeneous full-screen projection with varying red pixel values. Independent tests will be performed to estimate the range of laser power required for significant photo activation of the opsin and adjusted as necessary for each experimental mouse (Fig 2C).

Based on the preliminary experiments that we have performed, it appears that Jaws-mediated inhibition is effective in significantly reducing neuronal activity. However, if this proves to be insufficiently strong or if Jaws expression is not widespread enough, we will employ, as a back-up plan, stGtACR2, a blue-sensitive anion-conducting channelrhodopsin [51] that we have successfully employed in previous experiments. In this case, retinotopic mapping and the identification of the portions of V1 responsive to the Gabor patches will be done before the injection of the viral vector mediating the expression of stGtACR2, to prevent that the opsin-mediated inactivation will mask calcium signals (in view of the similar absorption spectrum of stGtACR2 and GCaMP6).

### Control experiments

#### Control experiment 1: Optogenetic controls .

In a subset of recording sessions we will perform experiments while illuminating a non-visual area (e.g., primary somatosensory cortex) in which no opsin expression is expected. All experimental conditions will be replicated to control for any confounding effect of photo-illumination. Analogously, in a subset of recording sessions (n >= 3) we will perform experiments while completely inactivating V1 – rather just the non-visually-responsive portion of V1. This will be important to verify the necessary role of V1 for performing the center of mass task.

#### Control experiment 2: Optogenetic controls.

Additional control experiments will be done to examine the possible role of the superior colliculus in the center of mass task. In this set of experiments, an injection of fluorescent muscimol will be done targeting the superior colliculus (max 200 nl), and a behavioral session will then be acquired. Animals will be sacrificed immediately afterwards for histological inspection. A negative result would indicate that the center of mass task is most likely solved by cortical areas. Conversely, if this control experiment reveals a functional role for the superior colliculus in solving the center of mass task (as indicated by a change in the psychometric response curves), we will revise the task in a way that limits the role of this subcortical structure. For instance, this can be achieved by replacing Gabor patches with more complex types of images whose processing is known to require cortical areas [52].

### Sequence of experimental activities

Following the completion of training, we will run experiments during a period of time when opsin expression should be optimal (between 3 and 8 weeks after viral injections). We plan to collect multiple sessions from each animal, so that at least one good session per activity level can be collected from each animal. As some exclusion criteria can only be assessed a posteriori, we will collect as many sessions as feasible from each animal. Furthermore, in this phase we will also test that mice can correctly respond to Gabor patches presented anywhere in the visual field. This will be done by presenting single Gabor patches; in this configuration, the center of mass corresponds to the individually presented patch.

At the start of the recording period in each mouse, we will perform a retinotopic mapping that will be used to calibrate patterned photo-illumination. This procedure will be repeated at least once – typically at the end of the recording period (but before the start of electrophysiological recordings, that may damage the PDMS cranial window) to verify the stability of the retinotopic mapping.

Following retinotopic mapping, each subsequent behavioral session will be done with a single visual background, and will be assigned a priori to the corresponding activity level. The order in which backgrounds are used will be counterbalanced across mice. Pupil-size monitoring and the results obtained during both pilot experiments and recording sessions involving electrophysiological recordings will be used to group individual trials into the three different levels of background activity (MA, VI and NB conditions). In particular, we expect that trials will be mainly grouped based on which visual background is shown and on the level of arousal, as quantified by pupil diameter.

**Fig 5:**
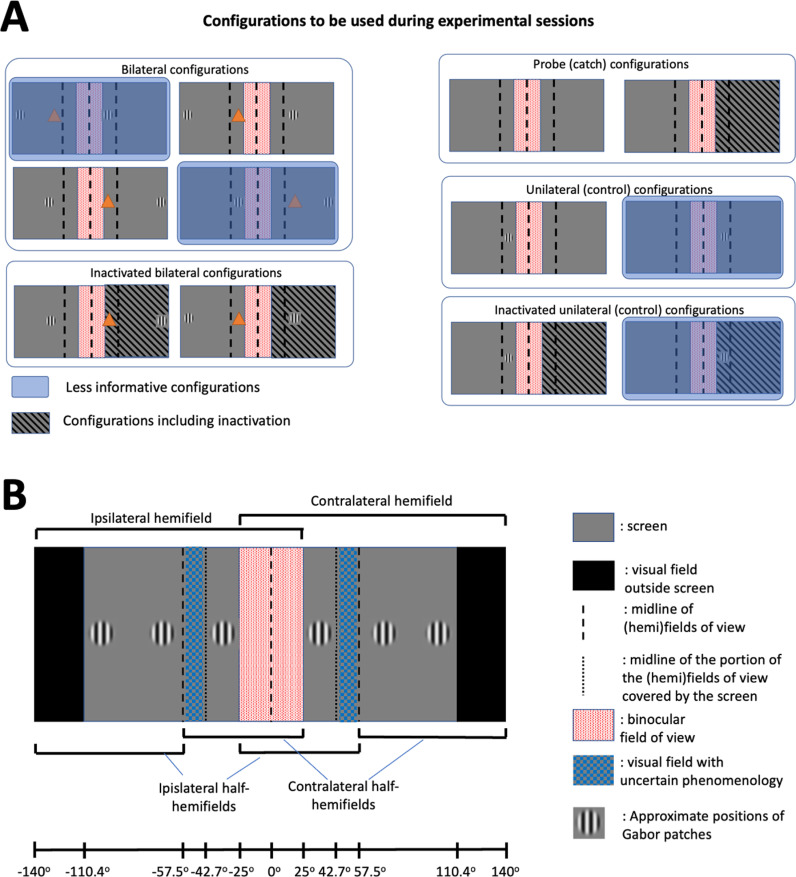
Experimental configurations. **A**. Various configurations of locations of the Gabor patches will be presented and coupled with optogenetic inactivation. Bilateral configurations will be used to answer to test the theories’ predictions (cf. Fig 6). In order to make the experiment feasible, we have selected a set of configurations for which the theories under scrutiny yield the most distinct predictions (cf. Fig 6). Importantly, some additional configurations (shaded in blue) are necessary to maintain a proper balance between stimuli rewarded with left vs. right licks – to prevent the insurgence of a response bias to either side. Two bilateral configurations will be coupled with optogenetic inactivation of background activity (here indicated with a striped shading). Probe trials will be used to estimate false alarm rates and any effect of optogenetic illumination on it. Finally, unilateral configurations will be used to test if optogenetic inactivation induces any bias in response side, in the absence of a stimulus being presented in the inactivated hemifield of view. Fractions and number of trials for each category are only an estimate that will depend on animals’ performance and on the actual number of trials in each experimental session. **B**. Diagram indicating the positions of the Gabor patches that will be used in the actual experimental sessions. Compared to the outline shown in Fig 1, we will have to adjust the locations where the Gabor patches are shown based on the actual coverage of the visual field by the screen where visual stimuli are displayed within the experimental setup. Specifically, while the visual field extends up to 140 deg beyond the midline, the screens only reach 110 deg. This introduces a possible confounding factor. Specifically, the line splitting each hemifield of view in half (dashed line) does not overlap with the line splitting the portion of the screen visible within each hemifield of view in two (dotted line). Under the assumption that optogenetic inactivation will induce a hemineglect-like deficit, we cannot predict if mice will respond based on the position of the still-perceived Gabor patch with respect to either the dashed or dotted line, i.e., based on the position of the Gabor on the screen or on the field of view. For this reason, to avoid possible confounding factors, we will avoid displaying Gabor patches in the portion of the visual field (shaded in blue) between these two lines, where no prediction can be made about the behavioral responses of mice.

**Fig 6:**
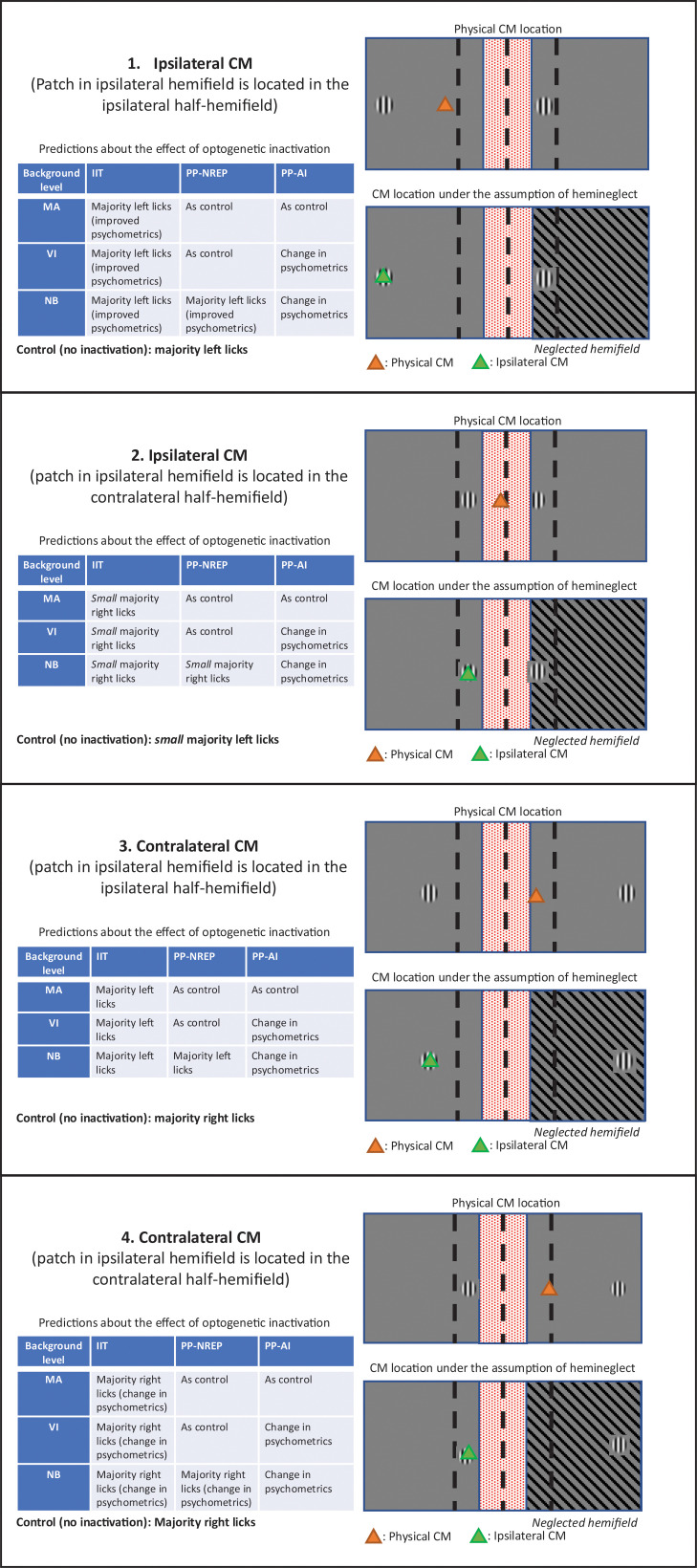
Scheme of experimental predictions. Various configuration of locations of the Gabor patches yield distinct experimental predictions, based on the level of background activity, and on whether inactivation of inactive neurons in V1 and HVAs in one hemisphere will induce hemineglect. Specifically, different predictions can be made based on whether the location of the center of mass (CM) for all Gabor patches falls in the contralateral or ipsilateral hemifield of view (with respect to the inactivated hemisphere), as well as on the position of the CM for the patches shown in the ipsilateral hemifield (i.e., based on whether this falls in the contralateral or ipsilateral half-hemifield - see also Fig 1A). Overall, four different configurations of stimuli will be tested (four panels in the figure). The same graphic conventions are used as in Figs 1 and 5. The area in the right hemifield with a striped background indicates the region of the visual field corresponding to the optogenetically manipulated portion of V1. Note that the regions where Gabor patches are shown correspond to portions of V1 that will not be optogenetically inactivated (see also Fig 1B). In this figure we assume that the left hemisphere is the inactivated one (hence: right is contralateral and left is ipsilateral). The “Control” label below each table indicates the expected behavior in the absence of optogenetic inactivation. MA: minimally active; VI: virtually inactive; NB: normal background; CM: center of mass; orange triangle: location of the CM for all shown patches; green: location of the phenomenologically experienced CM if optogenetic inactivation leads to functional hemineglect of the contralateral hemifield of view. The consequence of functional hemineglect (as predicted by either IIT or PP-NREP) is that mice will not experience visual space in the hemifield contralateral to the optogenetically inactivated hemisphere. Thus, the residual hemifield will be split into two half-hemifields, which will subjectively be experienced as the left and right hemifields. Therefore, in the presence of functional hemineglect, mice are expected to respond not based on the physical location of the center of mass but based on whether the ipsilaterally-presented Gabor patch (overlapping with the phenomenogically experienced CM) falls in either the left or right half-hemifield. Basically, IIT and NREP predict that, under some circumstances (different between IIT and NREP) that the total experienced space will shrink relative to the absolute space of the external visual field, and that mice will only respond based on the perceived location of the ipsilateral Gabor patch. Note that not all configurations are equally informative. For example, the predictions of IIT and PP-NREP for configuration 4 are similar and do not generally predict a major effect of optogenetic inactivation. In contrast, all theories have very different predictions on configuration 3. Therefore, only the most informative configurations will be used in the final experiments (cf. Fig 5A). Finally, while predictions are different for configuration 2, this configuration entails showing Gabor patches very close to the midline. We expect this configuration to be difficult for animals, hence we will not include it in the final experiment (cf. Fig 5A).

During each session, Gabor patches will be pseudo-randomly presented in a way yielding an equal number of trials with the center of mass of the two simultaneously-presented patches falling in one of 4 experimentally relevant locations (Fig 5A). These will always be displayed in the same position on the y axis (at eye level), and on a set of predetermined locations along the x axis (Fig 5B). These locations have been selected as they represent the stimulus configurations for which the predictions of the theories diverge the most (cf. Fig 6), can be discriminated by animals, and are balanced in terms of where Gabor patches are shown and especially of the side to which the animals must respond. A fraction of the trials (randomly selected) will include optogenetic inactivation of the retinotopic locations not directly activated by visual stimuli (see Fig 1). In the absence of optogenetic inactivation, only correct responses will be rewarded. Instead, in order to prevent biasing mice towards a response strategy, all responses (i.e., both left and right licks) will be rewarded in trials with optogenetic inactivation. For this reason, only a fraction of trials can be coupled to optogenetic inactivation (max 1/3 of all trials), to prevent animals from incorporating this new rule into their repertoire. Finally, we will include a set of unilateral control conditions, in which we will only display one Gabor patch, as well as probe trials (Fig 5A). These conditions are required to verify the effect of optogenetic inactivation during the presentation of visual stimuli and not only during the center of mass task, and therefore determine to which extent optogenetic manipulations specifically affect performance during the center-of-mass task.

After at least two successful behavioral sessions per activity level have been completed, three control sessions involving either multi-area ensemble recordings (experimental lab) or two-photon calcium imaging (replication lab) will be done. These post-sessions will be performed to verify that, in each animal, the assumptions underlying the three different activity levels are satisfied.

#### Multi-area ensemble recordings – post-sessions .

1) In a first recording session (post-session #1), probes will be located in V1 and in cortical areas receiving direct monosynaptic projections from V1 such as HVAs, the premotor region ALM [53] – an area that receives strong projections from dorsomedial HVAs [54] – and the anterior cingulate cortex (ACC). We will then perform a behavioral session without optogenetics, while varying both stimulus location as well as visual background. This recording session will allow us to verify that, in each animal, the different types of visual background elicit a level of baseline activity compatible with MA, VI and NB levels, as well as how baseline activity varies in each mouse as a function of arousal. Importantly, different levels of background and sensory-evoked activity will be elicited in each recording session in the same recording locations: the three levels of spontaneous activity (MA, VI and NB), in the presence or absence of stimuli impinging on the receptive field of recorded neurons. For this reason, we will not determine the presence of inactive neurons only based on the absence of spikes, but also on the changes in activity across conditions. This will exclude that we may characterize a state as being MA not because neurons are not spiking, but because we are not recording activity (e.g., because of a probe malfunction).
2) In a second session (post-session #2), we will place one silicon probe in V1 and verify that targeted photoillumination (via a colocalized optic fiber) is effective in reducing baseline activity levels for neurons not directly activated by visual stimuli in MA, VI and NB conditions. To achieve this, we will show only background visual stimuli while performing recordings and optogenetic inactivation.
3) In a third recording session, a probe will target a downstream cortical area receiving direct monosynaptic projections from V1 such as ACC, and we will perform optogenetic inactivation of V1 as a function of background activity level (MA, VI and NB). Also, in this session we will only display background stimuli. This last session (post-session #3) will be done to verify that inactivation of neurons firing at MA and VI baseline levels does not modulate downstream activity.

#### Two-photon calcium imaging – post sessions.

In the replication lab, the three sessions planned to be done after behavioral acquisition will be done using instead two-photon imaging. This will allow to exploit and combine the features of both electrophysiological recordings and calcium imaging (single-spike resolution in electrophysiology vs. ability to identify non-spiking neurons with two-photon calcium imaging). Post-session #1 will be done via performing two-photon imaging in V1 during a regular behavioral session; this will allow us to study the spatial architecture of V1 responses around the areas retinotopically corresponding to the presented Gabor patches. Specifically, imaging will be performed through the PDMS window on visual cortices while the animals are shown visual stimuli with different background levels, to confirm that each level elicits the expected background activity – when also taking into account neurons not spiking or with a very low firing rate, that cannot be detected with electrophysiological recordings. For post session #2, V1 neurons determined to be not responsive to the Gabor patches will be inactivated via two-photon targeted optogenetics [9]. In detail, imaging will be performed in visual cortices while the mouse is shown the same Gabor patches used in the experimental conditions. Recorded data will be immediately analyzed to determine whether single neurons are tuned to the presented visual stimuli. Non-tuned cells will be selected in random sets of up to 80 cells and selectively inactivated during a subsequent presentation of the Gabor patches. This will allow us to determine the effect inhibition of non-tuned cells on the tuned cells. Finally, post session #3 will involve widefield optogenetic inactivation of V1 and two-photon imaging of a downstream cortical area (e.g., ACC). Thus, we will opto-inhibit visual areas at the different levels of background activity to confirm that inactivation of spontaneous activity at the MA and VI levels does not affect downstream activity. Furthermore, this form of replication will ensure that the results that will be obtained will not depend on the acquisition modality being used (electrophysiology vs. two-photon calcium imaging).

### Histology

At the end of experiments, mice will be anesthetized with pentobarbital and sacrificed by transcardial perfusion (4% PFA in PBS). Brains will be sliced and imaging will be done to verify expression patterns of the opsins, probe location, and – if applicable – extent of muscimol diffusion.

### Planned analyses

The main outcome parameter of this experiment will be the analysis of behavioral performance as a function of position of the displayed Gabor patches and presence/absence of optogenetic inactivation. Specifically, the proportion of left vs. right licks, as well as misses, will be computed as a function of the horizontal position in the field of view of the center of mass of the presented Gabor patches. A d’ index will also be computed, based on a signal detection framework for multi-alternative behavioral tasks [55]. For trials with optogenetic inactivation, the same analysis will be done also considering the center of mass of stimuli presented in the ipsilateral field of view.

Widefield imaging data will be initially processed to extract the relevant neuronal activity [43]. Briefly, raw calcium responses will be motion corrected and denoised, and relative changes in fluorescence (dF/F) will be calculated with respect to the baseline period 2s before the presentation of visual stimuli. This dF/F will finally be corrected for overlapping hemodynamic signals by a linear regression procedure. Corrected responses to the mapping stimulus will be then used to generate azimuth and altitude position maps [42] and combined to generate a visual sign map that allows for the determination of V1 boundaries. Corrected responses to task stimuli will be used to determine the smallest task responsive area. The boundary between responsive/non-responsive areas is not expected to show a sharp transition (Fig 4B), so a thresholding procedure will be used to define it. The registered maps will be used to define the areas to be illuminated by the calibrated DMD projector in the inactivation experiment (Fig 4C-D). As discussed earlier, for the purpose of inactivation experiments, responsive areas will be extended with a closed border region with a width of about 250 μm to avoid inactivating the dendritic arborizations of stimulus-responsive neurons.

Analysis of multi-area ensemble recordings will focus on spiking activity. After spike sorting, the collected data using the Kilosort package [56], we will compute peri-stimulus time histograms (PSTHs) of single neurons as a function of stimulus location, to identify which neurons are directly activated by the presented Gabor patches (as described earlier [46]), and as a function of optogenetic onset, to investigate the effect of photo-stimulation.

Two-photon imaging data will be processed with a custom pipeline based on the CaImAn python library [57]. Briefly, raw data will be motion corrected and neuronal sources will be extracted, demixed and deconvolved using constrained non-negative matrix factorization (CNMF) and the OASIS deconvolution algorithm. Deconvolved activity will then be used to estimate neuronal firing rates by extracting the discrete first derivative, smoothed with a Gaussian kernel of 100–200 ms. Trial-averaged responses to visual or optogenetic stimuli will then be analyzed by calculating, on a cell-by-cell basis, the mean and standard deviation of the baseline activity during the 500 ms pre-stimulus window across conditions, and these parameters will be used to z-score the firing rates to compare activity across cells. Cell responsiveness will then be assessed to be present if evoked mean activity will be higher than a threshold of 1.67 standard deviations over baseline (equivalent to one tailed p-value of 0.05).

### Replication plan

Experiments will be simultaneously performed in two laboratories (Olcese lab, University of Amsterdam; Yuste lab, Columbia University). Experimental procedures have been jointly developed and implemented. Electrophysiological control recordings to verify that different visual backgrounds induce separate levels of background activity will only be performed in the Olcese lab. In the Yuste lab, the three sessions planned to be done after behavioral acquisition will be instead performed using two-photon imaging. Thus control sessions will reach the same objectives in the two labs, but will leverage the different properties of large-scale electrophysiology and imaging approaches [58]. This form of replication will allow us to test whether the results that will be obtained will not depend on the acquisition modality being used (electrophysiology vs. two-photon calcium imaging). Once data collection is completed, datasets will be separately analyzed, and results will only be compared at the end of the analysis procedure. Research personnel affiliated with the theory leads responsible for each of the three theories will also contribute to data analysis. A replication committee and personnel affiliated with the laboratories of the theory leads will verify that all data collection and analytical procedures have been correctly implemented and applied.

### Operational hypotheses and expected outcomes

The main outcome of the experiment will be reflected by measuring the effect of behavioral performance during the center of mass task following optogenetic inactivation of neurons not displaying sensory-evoked activity (cf. Fig 1B). Predictions will differ based on *a)* the positions of the Gabor patches with respect to the midline of the (hemi)fields of view (see also Fig 5) and *b)* the level of spontaneous activity (MA, VI and NB) displayed by neurons being inactivated. The relationship between the possible locations in which Gabor stimuli are presented, the level of spontaneous activity, and the predictions of the three theories under scrutiny as regards the potential effects of optogenetic inactivation of background activity are summarized in Fig 6

Specifically, Fig 6 discusses the outcomes expected by the different theories for all possible configurations of Gabor patches (when presenting one Gabor patch per hemifield of view). These configurations can all be produced with a fixed number of Gabor locations (Fig 5B). However, not all configurations lead to different predictions by the three theories, hence we only selected a subset in our experimental paradigm (Fig 5A).

Furthermore, in order to comprehend the different experimental outcomes predicted by IIT, NREP and AI-C, we point out that these theories have different predictions concerning the impact of optogenetic inactivation on perception. For both IIT and NREP, inactivating a certain fraction of V1 will lead to the disappearance of the portion of visual space retinotopically represented by the inactivated fraction of V1 (provided, in the case of NREP, that the inactivated neurons were sufficiently active before being inactivated) [11,13,18]. This would behaviorally correspond to a neglect of the corresponding portion of visual space. For AI-C, on the other hand, inactivating a fraction of V1 will not make the corresponding portion of visual space disappear from perception [33]. Instead, the confidence in the perceived visual information will vary, and consequently features of behavioral performance such as reaction time will be affected.

Importantly, the expected effect of optogenetic inactivation is not a complete change in behavioral strategy, but rather a shift in the psychometric response curve. For instance, in the condition depicted in configuration 3 of Fig 6, in the absence of optogenetic inactivation mice would show (for example) 70% licks towards the right spout (hits), 20% licks towards the left spout (errors) and 10% misses. Following optogenetic inactivation, under the assumption of a contralateral neglect (as predicted, e.g., by IIT), mice would shift to (for instance) 50% licks towards the left spout, 35% licks towards the right spout and 15% misses. Statistical comparisons will be done to assess whether a significant change in behavioral strategy has occurred. Specifically, we will compare (using either parametric or non-parametric tests (to be determined based on the distributions of the collected data) the d’, left-lick rate and right-lick rate in each of the relevant spatial configurations of visual stimuli (see Fig 5A).

Finally, each of the three theories not only makes a prediction about the effect of optogenetic inactivation of background activity on behavioral performance for the center-of-mass task, but also associates such prediction with a given level of confidence. For example, the prediction of IIT about the consequence of inactivating inactive neurons is a core prediction of the theory, and hence this is also a high-confidence prediction. Conversely, AI-C makes no specific, hard assumption about the role of background activity in perceptual experience. Consequently, the predictions of AI-C have a low confidence. Details are presented in Table 1 as regards experimental configuration 3 (Fig 6), which is the configuration with the highest power in discriminating between the three theories and the one on which we will focus our experiments (Fig 5A). Confidence will be used when determining to what extent experimental results either support or challenge each theory.

**Table 1:** Scheme of experimental predictions related to configuration 3 (see also Fig 6).

Minimally active neurons					
Predictions about the effect of inactivating Minimally Active neurons on % Left Licks					
Theory	Decrease	No change	Increase	Confidence	Rationale
IIT			X	High	Inactivation collapses the right hemifield of view, so the mouse licks toward the only target it sees.
NREP		X		High	Inactivating already inactive neurons should not make a difference (but see addendum on what “minimally active” means).
AI		X		Medium	Belief updating (and hence behavior) will be unaffected
**Virtually inactive neurons**					
**Predictions about the effect of inactivating Virtually Inactive neurons on % Left Licks **					
**Theory**	**Decrease**	**No change**	**Increase**	**Confidence**	**Rationale**
IIT			X	High	Inactivation collapses the right hemifield of view, so the mouse licks toward the only target it sees.
NREP		X		Medium	Some neural activity is present, output effects not completely clear, suppressing may have (modest) effect
AI		X		Medium	Belief updating (and hence behavior) will be unaffected
** Normal background activity**					
**Predictions about the effect of inactivating Normal Background Activity on % Left Licks**					
**Theory**	**Decrease**	**No change**	**Increase**	**Confidence**	**Rationale**
IIT			X	High	Inactivation collapses the right hemifield of view, so the mouse licks toward the only target it sees.
NREP			X	Medium	NREP predicts that background activity is important in this situation, but the amount of background activity and strength of suppression matter for the effect size.
AI	X		X	Low	Precision estimates may be altered, engendering an attentional bias towards one hemifield

## Discussion

The study presented in this protocol aims to test incompatible predictions made by three theories of consciousness (IIT, NREP and AI-C) about the role of spontaneous cortical activity in the generation of conscious experience. Specifically, we expect that the results will provide evidence supporting only one of the theories, thus challenging the other two. The experiment has been designed in close association between experimentalists and theoreticians, and the latter have openly committed to accepting the outcome of the experiment. Nevertheless, some possible confounding factors and limitations need to be taken into account.

First, we might not be able to reliably modulate spontaneous cortical activity such that all three different levels (MI, VI and NB) will be obtained. This might limit the extent to which the experiment will be able to provide evidence only supporting a theory but not the others. For example, IIT and NREP have different predictions for the MA and VI levels, but not for the NB level.

Second, the behavioral effect of optogenetic inactivation of the inactive portions of V1 might be less clear than expected, as a consequence of, for example, an incomplete inactivation of the area being illuminated. Control experiments have been planned to assess the effectiveness of optogenetics, but the possibility of obtaining null results cannot be completely discounted. Conversely, even an effective optogenetically induced hyperpolarization might also lead to confounding effects, as a consequence of the loss of lateral excitation to the portions of V1 still processing the visual stimuli (at least in the NB condition). We will control for this possibility through the unilateral stimulus configurations, for which we expect no effect of background inactivation on detection performance.

Finally, based on preliminary experiments, we considered it unfeasible to reliably identify and inactivate the inactive portions of both V1 and all HVAs at the same time. The latter, in fact, are much smaller than V1 and show coarser receptive fields that are not always clearly identifiable – in contrast to V1. It is thus possible that inactivating only the inactive portions of V1 might still allow HVAs, under IIT’s framework, to create a spatial experience for the portions of the visual field whose V1 representation is being inactivated. In fact, HVAs would still receive inputs from the non-inactivated and sensory-responsive portions of V1. Mice could therefore still experience the portion of the visual field contralateral to the inactivated hemisphere, albeit possibly at a coarser spatial scale [13]. To limit this potential issue, we will present mice with the smallest possible Gabor patches they can reliably perceive, i.e., at a spatial scale for which V1 is likely necessary, but HVAs are likely not.

The above mentioned factors indicate the importance of carefully assessing any null results we might obtain. These should not be taken as evidence either supporting or challenging a theory in the absence of additional (positive) evidence. Specifically, to address this issue, as well to provide an experimental framework capable of fully comparing key predictions of all three theories, this experimental protocol forms, together with three other protocols being separately published, a single unitary adversarial collaboration. The results of all experiments forming the collaboration will need to be jointly evaluated in order to be able to draw conclusions about the validity of the predictions of the three theories under scrutiny.

### The adversarial collaboration setting for this study

This study protocol forms part of a consortium project engaged in adversarial collaboration among three theories of consciousness: IIT, NREP and AI-C (https://doi.org/10.54224/20646). The results for the predictions described above, as well as the results from the other experiments in this adversarial collaboration, will be subject to discussion and comparative interpretation by the consortium, taking into account the overall set of predictions registered in protocols and at the website of the Open Science Foundation (OSF). Furthermore, we plan an integrative analysis according to the scheme set out in [59].

### Dissemination Plans

The outcomes of this experiment will likely be published as a stand-alone article in the first instance, or might be published as one paper combined with the outcome of the other experiments involved in the adversarial collaboration. Each of these experiments and their components may or may not be presented through posters and talks at conferences.

Following the completion and publication of the experiments described in each of the protocols published by the respective theory groups, an integrative analysis along the lines of ref. [59] may follow.

Finally, the INTREPID consortium will deliver at least one public talk or symposium to present and discuss the outcomes of the entire adversarial collaboration.

### Data management and sharing plan

All experimental protocols, metadata and data will be made available upon the completion of the project and publication of the related manuscripts via the EBRAINS open-access repository (https://ebrains.eu/). We will adhere to FAIR data principles and, whenever possible, we will use standard data formats such as Neuroscience Without Borders (NWB).

All code (Python, R and Matlab scripts) will be shared via GitLab or GitHub repositories.

### Dealing with Amendments

In the present experiment (as in the other protocols that form part of the INTREPID consortium), amendments will be considered and agreed upon between the experimental and replication teams, and the three theory leads (Karl Friston, Cyriel Pennartz, and Giulio Tononi). Upon the agreement of any reasonable amendments to the protocol, the amendment will be timestamped and added to the existing OSF preregistration documentation (https://osf.io/35rhx).
